# GABA_A_ Receptors in Astrocytes Are Targets for Commonly Used Intravenous and Inhalational General Anesthetic Drugs

**DOI:** 10.3389/fnagi.2021.802582

**Published:** 2022-01-11

**Authors:** Woosuk Chung, Dian-Shi Wang, Shahin Khodaei, Arsene Pinguelo, Beverley A. Orser

**Affiliations:** ^1^Department of Physiology, University of Toronto, Toronto, ON, Canada; ^2^Department of Anesthesiology and Pain Medicine, Chungnam National University, Daejeon, South Korea; ^3^Department of Anesthesiology and Pain Medicine, University of Toronto, Toronto, ON, Canada; ^4^Department of Anesthesia, Sunnybrook Health Sciences Centre, Toronto, ON, Canada

**Keywords:** astrocyte, GABA_A_ receptors, general anesthesia, etomidate, sevoflurane, perioperative neurocognitive disorders, patch-clamp

## Abstract

**Background:** Perioperative neurocognitive disorders (PNDs) occur commonly in older patients after anesthesia and surgery. Treating astrocytes with general anesthetic drugs stimulates the release of soluble factors that increase the cell-surface expression and function of GABA_A_ receptors in neurons. Such crosstalk may contribute to PNDs; however, the receptor targets in astrocytes for anesthetic drugs have not been identified. GABA_A_ receptors, which are the major targets of general anesthetic drugs in neurons, are also expressed in astrocytes, raising the possibility that these drugs act on GABA_A_ receptors in astrocytes to trigger the release of soluble factors. To date, no study has directly examined the sensitivity of GABA_A_ receptors in astrocytes to general anesthetic drugs that are frequently used in clinical practice. Thus, the goal of this study was to determine whether the function of GABA_A_ receptors in astrocytes was modulated by the intravenous anesthetic etomidate and the inhaled anesthetic sevoflurane.

**Methods:** Whole-cell voltage-clamp recordings were performed in astrocytes in the stratum radiatum of the CA1 region of hippocampal slices isolated from C57BL/6 male mice. Astrocytes were identified by their morphologic and electrophysiologic properties. Focal puff application of GABA (300 μM) was applied with a Picospritzer system to evoke GABA responses. Currents were studied before and during the application of the non-competitive GABA_A_ receptor antagonist picrotoxin (0.5 mM), or etomidate (100 μM) or sevoflurane (532 μM).

**Results:** GABA consistently evoked inward currents that were inhibited by picrotoxin. Etomidate increased the amplitude of the peak current by 35.0 ± 24.4% and prolonged the decay time by 27.2 ± 24.3% (*n* = 7, *P* < 0.05). Sevoflurane prolonged current decay by 28.3 ± 23.1% (*n* = 7, *P* < 0.05) but did not alter the peak amplitude. Etomidate and sevoflurane increased charge transfer (area) by 71.2 ± 45.9% and 51.8 ± 48.9% (*n* = 7, *P* < 0.05), respectively.

**Conclusion:** The function of astrocytic GABA_A_ receptors in the hippocampus was increased by etomidate and sevoflurane. Future studies will determine whether these general anesthetic drugs act on astrocytic GABA_A_ receptors to stimulate the release of soluble factors that may contribute to PNDs.

## Introduction

Astrocytes, which are among the most abundant cells in the mammalian brain, play an essential role in numerous functions, from the maintenance of molecular, cellular, and metabolic homeostasis to the regulation of cognition and behavior (Verkhratsky and Nedergaard, 2018; Santello et al., 2019). Not surprisingly, astrocytes are implicated in a variety of neurological disorders, including neurodegenerative diseases, ischemic stroke, epilepsy, and depression (Liu and Chopp, 2016; Wang et al., 2017; Santello et al., 2019; Siracusa et al., 2019; De Majo et al., 2020; Zhang et al., 2021).

One group of cognitive conditions that is of particular interest to the fields of anesthesia and critical care is perioperative neurocognitive disorders (PNDs) (Evered et al., 2018). PNDs occur most commonly in older patients after anesthesia and surgery. These patients may experience a range of symptoms, including delirium, confusion, inattention, and cognitive deficits, that can persist for days to months (Evered et al., 2018). The incidence of PNDs is remarkably high, ranging from 10 to 60%; and these disorders are associated with poor long-term outcomes, increased healthcare costs, loss of independence, and increased mortality (Witlox et al., 2010; Moskowitz et al., 2017; Sprung et al., 2017; Boone et al., 2020). Few effective prevention and treatment strategies are currently available (Berger et al., 2018; Mahanna-Gabrielli et al., 2019). Therefore, PNDs represent a major unmet health concern.

The causes of PNDs are complex and multifactorial, with general anesthetic drugs likely being one of several key contributing factors (Weinstein et al., 2018; Memtsoudis et al., 2019). Interestingly, we and others have postulated that astrocytes may play a causal role in PNDs (Terrando et al., 2013; Zurek et al., 2014; Wang et al., 2018; Li et al., 2020). Our previous studies using primary cultures of astrocytes, neurons, and astrocyte-neuron co-cultures have suggested that astrocytes contribute to the cognitive deficits that persist after brief exposure to general anesthetic drugs (Zurek et al., 2014; Wang et al., 2018). Indeed, we refer to these *in vitro* cell culture models that have demonstrated a crosstalk between astrocytes and neurons following exposure to anesthetic drugs as “PND in a dish.” Specifically, both an intravenous anesthetic drug (etomidate) and an inhalational agent (isoflurane) trigger a sustained increase in cell-surface expression and hence function of a subtype of γ-aminobutyric acid type A (GABA_A_) receptors in neurons (Zurek et al., 2014; Wang et al., 2018). Such an increase in GABA_A_ receptor function is sustained after the anesthetic drug is eliminated and is associated with long-lasting cognitive deficits (Zurek et al., 2014; Li and Zhang, 2021; Zuo et al., 2021). Furthermore, *in vitro* studies have shown that anesthetic drugs act on astrocytes to stimulate the release of one or more soluble factors that crosstalk with neurons, triggering a persistent increase in GABA_A_ receptor function in those neurons (Zurek et al., 2014; Wang et al., 2018). However, the receptors in astrocytes that act as targets for general anesthetic drugs have not yet been identified.

Astrocytes express a wide range of neurotransmitter receptors and transporters, including GABA_A_ receptors, which allow them to sense and respond to their surroundings (Verkhratsky and Nedergaard, 2018; Mederos and Perea, 2019). In contrast to what typically occurs in neurons, the activation of GABA_A_ receptors in astrocytes induces membrane depolarization, rather than hyperpolarization, and an increase in intracellular Ca^2+^ (Meier et al., 2008; Egawa et al., 2013; Mederos and Perea, 2019). These changes stimulate the release of various signaling molecules (Verkhratsky and Nedergaard, 2018). Because GABA_A_ receptors in neurons represent the primary target of most general anesthetic drugs (Garcia et al., 2010), GABA_A_ receptors in astrocytes may also be sensitive to commonly used drugs. These drugs may act upon GABA_A_ receptors in astrocytes to depolarize the membrane potential and trigger the release of soluble factors. Indeed, studies showing that pentobarbital, and the benzodiazepine agonists flunitrazepam and midazolam, increased the activity of GABA_A_ receptors in astrocytes were first reported in the 1980s and 1990s (Backus et al., 1988; Bormann and Kettenmann, 1988; Macvicar et al., 1989; Muller et al., 1994; Fraser et al., 1995). However, to date, no subsequent studies have directly examined the sensitivity of GABA_A_ receptors in astrocytes to modern general anesthetic drugs that are now in common use.

The goal of this study was to determine whether two representative general anesthetic drugs, etomidate and sevoflurane, modulate the function of astrocytic GABA_A_ receptors in hippocampal slices from mice. Etomidate is an intravenous agent that is often used for the induction of general anesthesia in critically ill patients because of its favorable hemodynamic profile (Hannam et al., 2019). Sevoflurane is one of the most commonly used inhalational anesthetic drugs (Brioni et al., 2017). Both these drugs have been shown to trigger the persistent increase in GABA_A_ receptor function in neurons (Zurek et al., 2014; Wang et al., 2018). Our results show that etomidate and sevoflurane increase the function of GABA_A_ receptors in astrocytes. These results provide the foundation for future studies, which will define the role of astrocytic GABA_A_ receptors in the pathophysiology of PNDs and assist in the development of potential new treatments for these disorders.

## Materials and Methods

### Experimental Animals

All experiments were performed with C57BL/6 male mice at postnatal days 21–27 (Charles River, Montreal, QC, Canada). This age was selected because astrocytes have reached maturity (Zhou et al., 2006), and the quality of brain slices significantly reduces with further aging (Lipton et al., 1995; Ting et al., 2014). Mice were housed in the animal care facility at the University of Toronto (Toronto, Ontario, Canada).

### Hippocampal Slice Preparation

Mice brains were obtained by decapitation after the mice were euthanized with a brief exposure to isoflurane. Sagittal brain slices (300 μm) containing hippocampus were prepared using a VT1200S vibratome (Leica, Deerfield, Illinois). Hippocampal slices were prepared in ice-cold sucrose-based cutting solution that contained (in mM): 212 sucrose, 25 NaHCO_3_, 5 KCl, 1.25 NaH_2_PO_4_, 10 glucose, 2 sodium pyruvate, 1.2 sodium ascorbate, 3.5 MgCl_2_, and 0.5 CaCl_2_. Slices were immediately transferred to a chamber containing artificial cerebrospinal fluid (aCSF) that contained (in mM): 125 NaCl, 25 NaHCO_3_, 2.5 KCl, 1.25 NaH_2_PO_4_, 10 glucose, 1.3 MgCl_2_, and 2.5 CaCl_2_. The slice chamber was first placed in a water bath (32°C, 30 min) for recovery of neuronal activities, and later placed at room temperature. All solutions were aerated with 95% O_2_/5% CO_2_ throughout the procedures.

### Whole-Cell Recordings of Astrocytes

Whole-cell patch-clamp recordings were performed at room temperature from astrocytes located in the stratum radiatum of the CA1 region of the hippocampus. Slices were transferred to a submersion recording chamber, where they were perfused with aCSF at 3–4 ml/min and were visualized using a 400x microscope (BX50WI; Olympus, Tokyo, Japan). Glass pipette resistance ranged between 3 and 5 MΩ. All recordings were performed using a MultiClamp 700B amplifier (Molecular Devices, Sunnyvale, California, United States), and data were acquired with pCLAMP 10.6 (Molecular Devices) via a Digidata 1550A interface (Molecular Devices).

Recordings were conducted with a KCl-based internal solution that contained (in mM): 140 KCl, 0.5 CaCl_2_, 1 MgCl_2_, 5 EGTA, 10 HEPES, 3 Mg^2+^-ATP (pH 7.3 using KOH at 290 mOsm). Upon achieving the whole-cell patch configuration, cells were confirmed as astrocytes based on the unique electrophysiological properties including a low membrane resistance (R_M_ < 15 MΩ), a low resting membrane potential (V_M_ < -70 mV), and a unique linear I-V relationship (Zhou et al., 2006, 2009; Du et al., 2016). The R_M_ and Ra were measured with “membrane test” protocol that is built into the pCLAMP 10.6 software (Molecular Devices). The resting membrane potential was measured in “I = 0” mode. The I-V relationship was tested by measuring currents that were generated in response to voltage steps from holding potentials that ranged from −180 to 0 mV, in 20 mV increments. Astrocytes were then voltage-clamped at their resting membrane potentials.

All slices were continuously perfused with aCSF that contained TTX (tetrodotoxin, 0.5 μM), APV ([2R]-amino-5-phosphonovaleric acid, 20 μM), and CNQX (6-Cyano-7-nitroquinoxaline-2,3-dione, 10 μM). Only recordings with an initial Ra less than 25 MΩ that varied less than 20% throughout the experiments were included in the analyses.

### Drugs and Chemicals

TTX was purchased from Alomone Labs (Jerusalem, Israel). APV and CNQX were obtained from Hello Bio Inc. (Princeton, NJ, United States). GABA and picrotoxin were from Sigma–Aldrich (Oakville, ON, Canada), while etomidate was purchased from US Pharmacopeia (Rockville, MD, United States) and sevoflurane was obtained from Abbott Laboratories (North Chicago, IL, United States).

Stock solutions of etomidate (100 mM) were prepared by dissolving etomidate powder in propylene glycol (35% v/v in physiological saline) and were stored at 4°C (Sprung et al., 2000). A final concentration of etomidate at 100 μM was used for the studies. Sevoflurane (532 μM) was diluted from the saturated aqueous phase of sevoflurane and was prepared at room temperature, as previously described (Lecker et al., 2013). This concentration of sevoflurane is twice the MAC (Minimum Alveolar Concentration of anesthetics) value for sevoflurane and was selected to ensure adequate drug levels in the slices (Nishikawa and MacIver, 2001; Lecker et al., 2013). In brief, 50 ml of sevoflurane was mixed with 100 ml of aCSF in a gas-tight glass bottle and stored at 4°C overnight. Sevoflurane at the saturated aqueous phase was measured at 11.8 mM (Lecker et al., 2013).

### GABA Puff Application

Focal puff applications of GABA (300 μM) were applied to the soma of the astrocytes using a Picospritzer system (Picospritzer II, Parker Hannifin, United States). A glass pipette (tip diameter 3–5 μm) that was filled with aCSF containing GABA (300 μM) was placed approximately 50–100 μm away from the cell soma before performing whole-cell configuration. The concentration of GABA was chosen based on a previous study (EC_50_ = 300 μM) (Ma et al., 2012). Puff pressure was set at 15–20 psi, and puff duration between 20 and 150 ms to obtain a baseline current amplitude of approximately 50–100 pA.

### Data and Statistical Analyses

The peak amplitude, rise time, decay time, and area of GABA-evoked responses were analyzed with Clampfit 10.7 software (Molecular Devices). The rise time was measured as the time from 10 to 90% of peak amplitude, and the decay time was defined as the duration from 90 to 40% of peak amplitude due to fluctuations in late decay phase. The area of the current responses was measured to the point where currents returned to baseline.

Data are represented as mean ± SD (Standard Deviation). Statistical analyses were performed using R statistical software version 3.6.1 (R Foundation for Statistical Computing, Vienna, Austria). All continuous variables were tested to determine whether they met conditions of normality (Shapiro-Wilk test) and homogeneity of variance (Levene’s test). Paired Student’s *t*-test was performed to compare paired data. A two-tailed hypothesis test was used, and statistical significance was set at *P* < 0.05.

## Results

### Identification of Astrocytes in Hippocampal Slices

**FIGURE 1:**
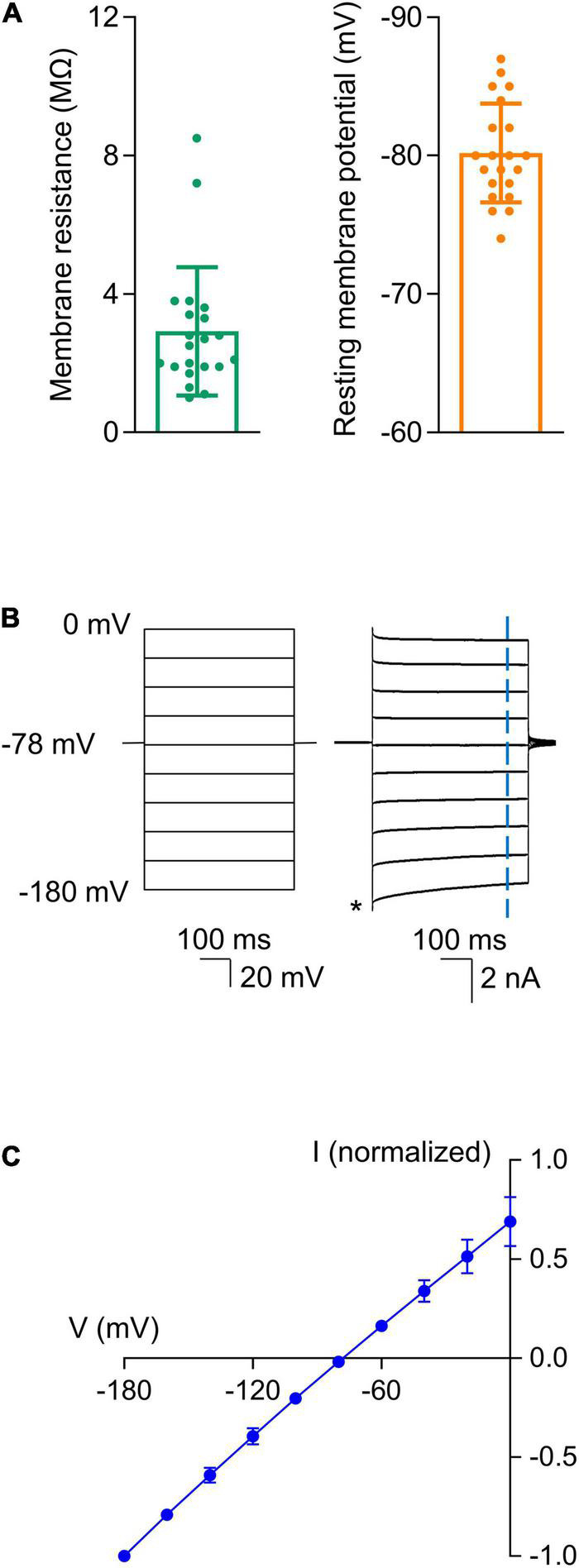
Astrocytes displayed unique electrophysiological properties. After the whole-cell patch configuration was achieved, cells were confirmed to be astrocytes based on their low membrane resistance, hyperpolarized resting membrane potentials, and linear I-V relationships. **(A)** Summarized data for membrane resistance and resting membrane potential (*n* = 21). **(B)** Linear I-V relationship of the astrocytes. Representative recordings show current responses (Right) to voltage steps (500 ms) that ranged from -180 to 0 mV in 20 mV increments (Left). The cell was held at -78 mV between voltage commands. Current amplitudes were measured 435 ms after initiation of each step voltage as indicated by the vertical blue dash line. The amplitudes were normalized to that at -180 mV as indicated by the asterisk. **(C)** Summarized I-V plot. Each data point represents the mean of values from 21 recorded astrocytes. The reversal potential obtained from the fitted I-V plot was -78 mV. Data are presented as mean ± SD.

Astrocytes were identified in hippocampal slices based on their morphology and electrophysiological properties. We recorded from cells that were relatively small, as astrocytes have a diameter of approximately 10 μm with round or irregularly shaped somas (Zhou et al., 2006; Du et al., 2015). Morphology alone was not sufficient to identify the astrocytes as other cell types, such as interneurons, have similar structural properties (Zhou et al., 2006). Thus, we next examined the electrophysiological properties of each cell to confirm that the recorded cells were indeed astrocytes. Astrocytes have unique electrophysiological properties that allow them to be readily distinguished from other cell types including interneurons. Specifically, astrocytes have a low membrane resistance (R_M_ < 15 MΩ), a relatively hyperpolarized resting membrane potential (V_M_ < -70 mV), and they generate a linear current-to-voltage (I-V) relationship in response to voltage steps due to passive K^+^ membrane conductances (Zhou et al., 2006, 2009; Ma et al., 2014; Du et al., 2016). All the cells we recorded from had a low membrane resistance (3.2 ± 2.2 MΩ, *n* = 21) and a hyperpolarized resting membrane potential (−80.2 ± 3.7 mV, *n* = 21; Figure 1A). The cells also displayed a linear I-V relationship in response to voltage steps (−180–0 mV in 20 mV increments), as shown in Figures 1B,C.

### GABA Activates γ-Aminobutyric Acid Type A Receptors in Astrocytes

**FIGURE 2:**
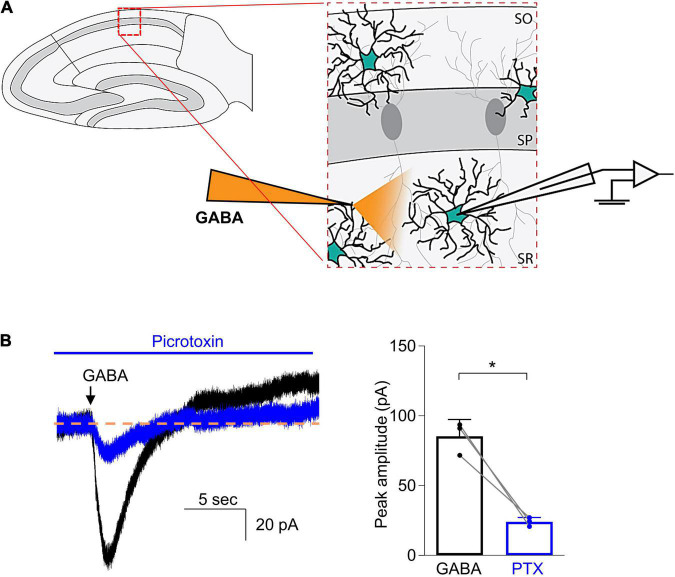
Focal puff application of GABA to astrocytes induced an inward current that was inhibited by picrotoxin. **(A)** Schematic drawing shows that a glass capillary containing GABA (300 μM) was placed 50–100 μm away from the recorded astrocyte in the stratum radiatum of CA1 region of a hippocampal slice. **(B)** Left: Representative traces demonstrate current responses to puff applications of GABA before (black) and during addition of picrotoxin (0.5 mM, blue), a non-competitive GABA_A_ receptor antagonist. Right: Quantified data show the inhibitory effects of picrotoxin (PTX). *n* = 3, *P = 0.019, paired Student’s *t*-test. Data are presented as mean ± SD.

To study the effects of general anesthetic drugs on the function of GABA_A_ receptors in astrocytes, we first needed to record stable GABA-evoked responses. We used focal puff applications of GABA as previous studies have examined GABA_A_ receptor-dependent currents using similar methods (Ma et al., 2012). A glass capillary that contained GABA (300 μM) was placed 50–100 μm away from the recorded cell (Figure 2A). After the whole-cell patch configuration was successfully established in astrocytes, a focal puff application of GABA was applied. The application of GABA consistently activated an inward current (Figure 2B). To confirm the GABA-evoked responses were generated by GABA_A_ receptors, the non-competitive GABA_A_ receptor antagonist picrotoxin (0.5 mM) was then added to the bath solution for 5–10 min and a subsequent puff of GABA was applied. The amplitude of the peak current was measured before and during the application of picrotoxin. The peak current was reduced to 28.9 ± 8.0% of the control (Figure 2B, 85.4 ± 12.0 pA for GABA vs. 24.0 ± 3.2 pA for GABA + picrotoxin, n = 3; *P* = 0.019, paired *t*-test). These results showed that the inward currents evoked by GABA were primarily generated by GABA_A_ receptors.

### Etomidate Increases the Function of γ-Aminobutyric Acid Type A Receptors in Astrocytes

**FIGURE 3:**
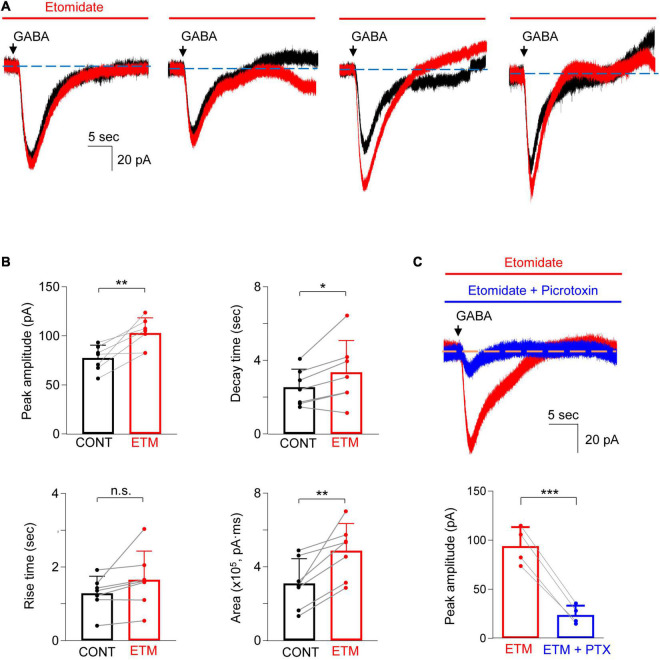
Etomidate potentiated GABA-evoked current in astrocytes. **(A)** Representative traces show the GABA-evoked responses before and during etomidate (ETM) treatment from four different astrocytes. Note the different shapes of the late phase of the current decay in both the absence and the presence of etomidate. **(B)** Summarized data for peak amplitude, rise time, decay time, and charge transfer (area). *n* = 7, *P < 0.05, **P < 0.01, n.s.: not significant, paired Student’s *t*-test. CONT: control. **(C)** Representative traces (top) and summarized data (bottom) show that GABA-evoked current in the presence of etomidate (ETM) is inhibited by picrotoxin (PTX, 0.5 mM, 5–10 min). *n* = 4, ***P = 0.001, paired Student’s *t*-test. Data are presented as mean ± SD.

We next sought to examine the effects of etomidate (100 μM) on GABA_A_ receptor-generated currents in astrocytes. After obtaining an initial baseline current in response to GABA (300 μM), the slices were perfused for 2 min with aCSF containing etomidate and a second puff of GABA was applied. The amplitude of the peak current (pA), the rise time and decay time (s) of the current, and the area (pA4⋅ms) under the current response curve were measured. Notably, we observed that the late decay phase of current evoked by GABA, both in the absence and the presence of etomidate, was somewhat unstable. Specifically, the decay current had several different undulating shapes, as shown in Figure 3A. This variability in the late phase of current decay differed from current recorded in hippocampal neurons, as observed by us and others (Bai et al., 1999; Caraiscos et al., 2004; Domínguez et al., 2016). To minimize the impact of the baseline instability on the analysis of current responses, the decay time of the current was measured from 90 to 40% of the peak amplitude.

Etomidate increased the amplitude of the current by 35.0 ± 24.4% (Figure 3B; control: 77.5 ± 13.0 pA, vs. etomidate: 102.9 ± 15.4 pA, *n* = 7; *P* = 0.006, paired *t*-test). Etomidate did not alter the current rise time (control: 1.3 ± 0.5 s vs. etomidate: 1.7 ± 0.8 s, *n* = 7; *P* = 0.10, paired *t*-test) but prolonged the decay time by 27.2 ± 24.3% (control: 2.6 ± 1.0 s vs. etomidate: 3.4 ± 1.7 s, *n* = 7; *P* = 0.037, paired *t*-test). The total charge transfer was also increased by 71.2 ± 45.9% [control: 3.1 ± 1.4 (×10^5^) pA⋅ms vs. etomidate: 4.9 ± 1.5 (×10^5^) pA⋅ms, *n* = 7; *P* = 0.006, paired *t*-test]. We next confirmed that GABA-evoked responses in the presence of etomidate were primarily generated by GABA_A_ receptors. The co-application of etomidate and picrotoxin (0.5 mM) to the bath solution showed that the peak current was reduced by 75.3 ± 5.5% (Figure 3C; pre-picrotoxin: 94.0 ± 19.4 pA vs. picrotoxin: 23.8 ± 9.6 pA, *n* = 4; *P* = 0.001, paired *t*-test).

### Sevoflurane Increases the Function of γ-Aminobutyric Acid Type A Receptors in Astrocytes

**FIGURE 4:**
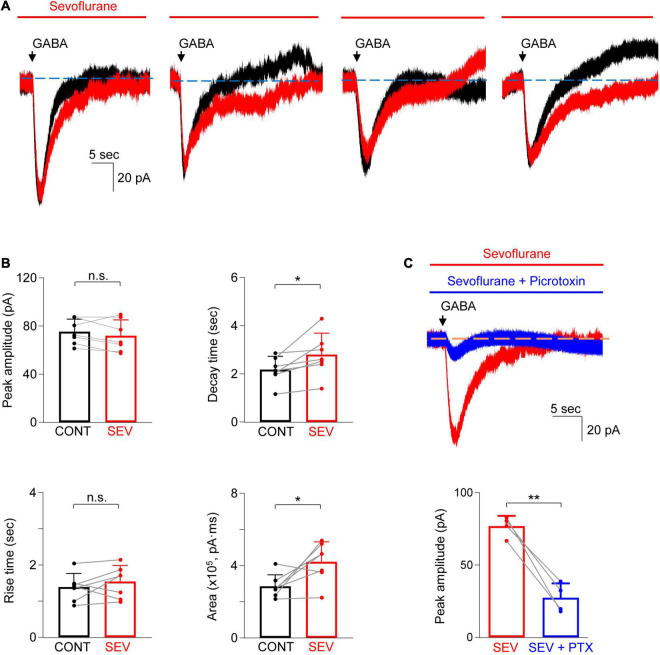
Sevoflurane potentiated GABA-evoked current in astrocytes. **(A)** Representative traces show the GABA-evoked responses before and during sevoflurane (SEV) treatment from four different astrocytes. Note the different shapes of the late phase of the current decay in both the absence and presence of sevoflurane. **(B)** Summarized data for peak amplitude, rise time, decay time, and charge transfer (area). *n* = 7, *P < 0.05, n.s.: not significant, paired Student’s *t*-test. CONT: control. **(C)** Representative traces (top) and summarized data (bottom) show that GABA-evoked current in the presence of sevoflurane (SEV) is inhibited by picrotoxin (PTX, 0.5 mM, 5–10 min). *n* = 4, **P = 0.003, paired Student’s *t*-test. Data are presented as mean ± SD.

In the next set of studies, sevoflurane (532 μM) was added to the bath solution and the changes in the peak amplitude and time course of GABA-evoked currents were investigated (Figure 4A). Sevoflurane did not increase the peak current (Figure 4B; control: 75.3 ± 10.4 pA vs. sevoflurane: 71.9 ± 10.4 pA, *n* = 7; *P* = 0.21, paired *t*-test) nor did it increase the current rise time (Figure 4B; control: 1.4 ± 0.4 s vs. sevoflurane: 1.5 ± 0.4 s, *n* = 7; *P* = 0.33, paired *t*-test). However, sevoflurane prolonged the current decay by 28.3 ± 23.1% (Figure 4B; control: 2.2 ± 0.6 s vs. sevoflurane: 2.8 ± 0.9 s, *n* = 7; *P* = 0.030, paired *t*-test) and increased the total charge transfer by 51.8 ± 48.9% [Figure 4B; control: 2.86 ± 0.64 (×10^5^) pA⋅ms vs. sevoflurane: 4.22 ± 1.10 (×10^5^) pA⋅ms, *n* = 7; *P* = 0.029, paired *t*-test]. Thus, sevoflurane increased the GABA-evoked currents. GABA-evoked currents in the presence of sevoflurane were also inhibited by 64.4 ± 12.5% when picrotoxin (0.5 mM) was added to the bath solution (no picrotoxin: 76.9 ± 7.1 pA vs. picrotoxin: 27.4 ± 10.0 pA, *n* = 4; *P* = 0.003, paired *t*-test), confirming that the currents were mainly GABA_A_ receptor-dependent (Figure 4C).

## Discussion

The goal of this study was to determine whether GABA_A_ receptors in astrocytes are targets for etomidate and sevoflurane. We first recorded GABA_A_ receptor–mediated current from astrocytes in the stratum radiatum of the CA1 region of hippocampal slices from mice and then showed that both etomidate and sevoflurane increased the GABA-evoked responses. More specifically, etomidate increased the peak amplitude of the current and prolonged its decay, whereas sevoflurane prolonged current decay but had no effect on the peak. Overall, both etomidate and sevoflurane increased the total charge transfer of the GABA-evoked responses. To the best of our knowledge, these results provide the first direct evidence that commonly used general anesthetic drugs increase the function of GABA_A_ receptors in astrocytes.

As noted by others, it is technically challenging to perform voltage-clamp recording of GABA-evoked responses in astrocytes (Ma et al., 2014). While patch-clamp recording techniques have been widely used by us and others to study anesthetic modulation of GABA_A_ receptors in neurons (Orser et al., 1994; Bai et al., 1999; Caraiscos et al., 2004; Schools et al., 2006; Zhou et al., 2021), astrocytes have a low membrane resistance because of passive K^+^ conductances and are extensively coupled into a syncytium through gap junctions (Ma et al., 2016), which makes patch-clamp recording difficult. Such unfavorable patch-clamp recording conditions cause the voltage change to occur primarily at the tip of the recording electrode, rather than across the cell membrane (Ma et al., 2014). Poor voltage-clamp conditions also cause instability of baseline currents and prevent accurate measurement of current responses during long-term whole-cell recordings. To minimize the impact of such current instability, we recorded from slices obtained from young mice and used a Picospritzer perfusion system to focally and rapidly deliver a transient puff (<150 ms) of GABA. Using this combined approach, we were able to record relatively stable GABA-evoked responses that were generated by GABA_A_ receptors.

Our results provide convincing evidence that the function of astrocytic GABA_A_ receptor is increased by commonly used general anesthetic drugs. These results are consistent with findings from several earlier studies that date back to the 1980s and 1990s of pentobarbital and benzodiazepines (Backus et al., 1988; Bormann and Kettenmann, 1988; Macvicar et al., 1989; Muller et al., 1994; Fraser et al., 1995). For instance, pentobarbital but not diazepam increased GABA-evoked responses in Bergmann glial cells of cerebellar slices from young animals (postnatal days 5–12) (Muller et al., 1994). However, these studies were undertaken at a development stage when glial cells are still immature (Zhou et al., 2006). In another study, using hippocampal slices, to which kainic acid had been applied to reduce the number of neurons, GABA-evoked responses in CA3 astrocytes increased after pentobarbital and flunitrazepam treatment (Macvicar et al., 1989). Although kainic acid helps in isolating astrocytes, it also modifies the surviving cells into a pathological state (Kim et al., 2007). The same limitation remains for acutely isolated astrocytes obtained through enzymatic digestion (Fraser et al., 1995). Surprisingly, since those earlier studies, little progress has been made regarding anesthetic modulation of astrocytic GABA_A_ receptors, probably because of the technical challenges outlined above. We overcame these limitations by recording currents in mature astrocytes in brain slices from young mice.

Interestingly, we observed that etomidate, but not sevoflurane, increased the peak amplitude of GABA responses in astrocytes. At least three factors could contribute to this difference including drug bioavailability, the effects of the drugs on GABA_A_ receptor kinetics, and the subunit composition of the underlying receptors. For instance, it is unlikely that “MAC equivalent” concentrations of etomidate and sevoflurane were present at the tip of the recording electrode as the physical properties of the drugs differ. Etomidate is stable in aqueous solution but requires a considerable time to penetrate the brain slices. Indeed, it can take as long as 1–2 h to reach an equilibrium in brain slices (Benkwitz et al., 2007). In contrast, sevoflurane readily diffuses into brain tissues, but rapidly evaporates from the perfusion solution (Nishikawa and MacIver, 2001; Sebel et al., 2006). Another reason could be differences in drug action on GABA_A_ receptor kinetics. Anesthetic drugs generally increase the potency of GABA, increase the rate of receptor activation, and slow the rate of receptor deactivation (Orser et al., 1994; Yang and Uchida, 1996; Belelli et al., 1997; Bai et al., 1999; Benkwitz et al., 2004). However, etomidate, similar to other intravenous anesthetics including propofol, reduced desensitization, whereas volatile anesthetics increased receptor desensitization (Wu et al., 1996; Liu et al., 2015). Since the peak current reflects the summed effects of receptor activation, deactivation and desensitization, differences in the peak current could result from differences in drug action on receptor kinetics. Finally, the effects of anesthetic drugs on GABA_A_ receptor kinetics are highly dependent on the subunit composition of the receptors. Thus, the subunit composition of the heterogeneous GABA_A_ receptors influences the response to anesthetic drugs (Uchida et al., 1995; Krasowski et al., 1998; Jenkins et al., 2001; Nishikawa and Harrison, 2003; Benkwitz et al., 2004; Zhong et al., 2008; Hoft et al., 2014; Woll et al., 2018; Liao et al., 2019).

Our results raise some interesting questions that are worthy of future studies. It would be of interest to examine the concentration-dependence of anesthetic modulation of astrocytic GABA_A_ receptors and whether the anesthetic sensitivity of astrocytic and neuronal GABA_A_ receptors differ. Given technical challenges with standard whole-cell patch-clamp recordings from astrocytes, we investigated the effects of just one anesthetic concentration with one concentration of GABA. A previous study overcame some of these technical challenges by using a dual-patch technique that permitted simultaneous recordings of membrane currents and potentials in astrocytes (Ma et al., 2014). Future studies might also investigate GABA_A_ receptors in astrocytes that are mechanically isolated from brain slices or macro patches that are excised from astrocytes. These techniques allow drugs and agonists to be rapidly applied to and then washed away from the recorded astrocytes. Drug concentrations can also be more accurately controlled to study the concentration-dependent effects of anesthetic drugs. Thus, the approaches allow anesthetic effects on GABA_A_ receptor responses in astrocytes and neurons to be more effectively compared.

Another important issue that requires further study is whether astrocytic GABA_A_ receptors play a role in PNDs *in vivo*. Given that exposure of astrocytes to anesthetic drugs triggers a sustained increase in cell-surface expression and function of GABA_A_ receptors in neurons *in vitro*, it is possible the drugs act upon astrocytic GABA_A_ receptors to cause similar changes *in vivo*. Indeed, general anesthetic drugs increase Ca^2+^ signaling in astrocytes by activating GABA_A_ receptors *in vivo* (Meier et al., 2008; Thrane et al., 2012). This increase in cytosolic Ca^2+^ may trigger the release of soluble factor(s) that modify the function of neighboring neurons. Future studies will determine whether astrocytic GABA_A_ receptors contribute to this crosstalk with neuronal GABA_A_ receptors that may contribute to PNDs, as well as identify the soluble factor(s). Using genetic approaches, such as astrocyte-specific gene knockdown/knockout, and novel CRISPR-based technology to target anesthetic-sensitive astrocytic GABA_A_ receptors *in vitro* and *in vivo*, may help answer these questions (Mori et al., 2006; Shinohara et al., 2016; Meneghini et al., 2021). Such studies may lead to the discovery of novel strategies to mitigate the cognitive dysfunction experienced by older patients with PNDs.

This study had some limitations. We were able to test only the early phase of current decay (90–40% decay time) induced by puff applications of GABA in astrocytes because the late phase is highly variable in both the absence and presence of anesthetic treatment. Such variability could be due to low membrane resistance of astrocytes and to changes in membrane resistance caused by secondary inhibition of the K^+^ channel after application of GABA (Ma et al., 2012, 2014). As noted above, performing dual-patch recordings in hippocampal astrocytes may reduce this variability and may help in further evaluating the effects of anesthetics on astrocytic GABA_A_ receptors. In addition, this study focused on the effects of anesthetics in mature hippocampal astrocytes at a single age of animal. However, a distinct feature of astrocytes is their heterogeneity across different brain regions (Matias et al., 2019; Batiuk et al., 2020). Also, the subunit compositions and expression profiles of GABA_A_ receptors are heterogeneous across brain regions and at different developmental stages (Backus et al., 1988; Macvicar et al., 1989; Muller et al., 1994; Fraser et al., 1995; Hoft et al., 2014; Zhang et al., 2016). Therefore, the anesthetic effects on GABA_A_ receptors in astrocytes may differ in different brain regions and at different ages.

In summary, etomidate and sevoflurane, two modern general anesthetic drugs used in clinical settings, increased GABA_A_ receptor function in hippocampal astrocytes. These results provide the foundation for future studies that will determine whether astrocytic GABA_A_ receptors contribute to PNDs.

## Data Availability Statement

The original contributions presented in the study are included in the article, further inquiries can be directed to the corresponding author.

## Ethics Statement

The animal study was reviewed and approved by the Animal Care Committee of the University of Toronto (Toronto, Ontario, Canada).

## Author Contributions

WC, D-SW, and BAO designed and developed the experiments and prepared manuscript. WC executed the experiments and analyzed the data. SK and AP helped in the design and development of experiments. All authors have approved the final manuscript.

## Conflict of Interest

BAO serves on the Board of Trustees of the International Anesthesia Research Society (San Francisco, California, United States) and was a co-director of the Perioperative Brain Health Centre (Toronto, Ontario, Canada; http://www.perioperativebrainhealth.com). She was a named inventor on a Canadian patent (2,852,978) and two U.S. patents (9,517,265 and 10,981,954). The new methods identified in the patents aim to prevent and treat delirium and persistent neurocognitive deficits after anesthesia and surgery, as well as to treat mood disorders. BAO collaborates on clinical studies supported by in-kind software donations from Cogstate Ltd. (New Haven, Connecticut, United States). The remaining authors declare that the research was conducted in the absence of any commercial or financial relationships that could be construed as a potential conflict of interest.

## Publisher’s Note

All claims expressed in this article are solely those of the authors and do not necessarily represent those of their affiliated organizations, or those of the publisher, the editors and the reviewers. Any product that may be evaluated in this article, or claim that may be made by its manufacturer, is not guaranteed or endorsed by the publisher.
